# Parotid gland-recovery after radiotherapy in the head and neck region - 36 months follow-up of a prospective clinical study

**DOI:** 10.1186/1748-717X-6-125

**Published:** 2011-09-27

**Authors:** Jeremias Hey, Juergen Setz, Reinhard Gerlach, Martin Janich, Guido Hildebrandt, Dirk Vordermark, Christian R Gernhardt, Thomas Kuhnt

**Affiliations:** 1 Department of Prosthetic Dentistry, University School of Dental Medicine, Martin-Luther-University Halle-Wittenberg, Halle, Germany; 2 Department of Radiotherapy, University Clinic, Martin-Luther-University Halle-Wittenberg, Halle, Germany; 3 Department of Operative Dentistry and Periodontology, University School of Dental Medicine, Martin-Luther-University Halle-Wittenberg, Halle, Germany; 4 Department of Radiotherapy, University Clinic, University Rostock, Rostock, Germany

**Keywords:** head and neck cancer, irradiation, saliva, hyposalivation, parotid gland sparing, recovery

## Abstract

**Background:**

The aim of the present study was to evaluate the recovery potential of the parotid glands after using either 3D-conformal-radiotherapy (3D-CRT) or intensity-modulated radiotherapy (IMRT) by sparing one single parotid gland.

**Methods:**

Between 06/2002 and 10/2008, 117 patients with head and neck cancer were included in this prospective, non-randomised clinical study. All patients were treated with curative intent. Salivary gland function was assessed by measuring stimulated salivary flow at the beginning, during and at the end of radiotherapy as well as 1, 6, 12, 24, and 36 months after treatment. Measurements were converted to flow rates and normalized relative to rates before treatment. Mean doses (D_mean_) were calculated from dose-volume histograms based on computed tomographies of the parotid glands.

**Results:**

Patients were grouped according to the D_mean _of the spared parotid gland having the lowest radiation exposure: Group I - D_mean _< 26 Gy (n = 36), group II - D_mean _26-40 Gy (n = 45), and group III - D_mean _> 40 Gy (n = 36). 15/117 (13%) patients received IMRT. By using IMRT as compared to 3D-CRT the D_mean _of the spared parotid gland could be significantly reduced (D_mean _IMRT vs. 3D-CRT: 21.7 vs. 34.4 Gy, p < 0.001). The relative salivary flow rates (RFSR) as a function of the mean parotid dose after 24 and 36 months was in group I 66% and 74%, in group II 56% and 49%, and in group III 31% and 24%, respectively. Multiple linear regression analyses revealed that the parotid gland dose and the tumor site were the independent determinants 12 and 36 months after the end of RT. Patients of group I and II parotid gland function did recover at 12, 24, and 36 months after the end of RT.

**Conclusions:**

If a D_mean _< 26 Gy for at least one parotid gland can be achieved then this is sufficient to reach complete recovery of pre-RT salivary flow rates. The radiation volume which depends on tumor site did significantly impact on the D_mean _of the parotids, and thus on the saliva flow and recovery of parotid gland.

## Background

Sparing salivary glands during radiotherapy (RT) is an important research field in the treatment of head and neck tumors because avoiding xerostomia or reduction of hyposalivation improves oral health and quality of life of the patients [1-3].

The functional changes of the parotid glands as well as the impact on oral structures depend on radiation dose and the irradiated volume [4]. Eisbruch *et al*. suggested that xerostomia could be avoided until a dose lower than 26 Gy [5]. Recently, a multicenter randomized study (PARSPORT trial) investigated the advantage of the parotid sparing of intensity-modulated radiotherapy (IMRT) technique as compared to conventional 3D-conformal-radiotherapy-technique (3D-CRT) in terms of clinical outcome [6]. The authors described that after 12 months, 39% of IMRT patients suffered from dry mouth as compared to 74% of conventional 3D-CRT patients. However, D_mean _values < 26 Gy of both parotid glands cannot be achieved in all patients even by using more advanced 3D-CRT or IMRT during uni- or bilateral radiotherapy in the head neck region. In addition, a functional recovery could be expected [7]. Moreover, most studies focusing on the recovery of the salivary glands after curative radiotherapy had only a follow-up period of 12 months. Just one single study by Braam *et al*. examined the quality of life and salivary flow rates after irradiation of head and neck cancer over a period of 5 years [8].

At the University Hospital of Halle, Germany, in the year 2002 an individualized 3D-CRT technique has been implemented in clinical practice to spare parotid glands [9]. Since 2007 the IMRT technique was implemented. The aim of this investigation was to measure of the whole salivary flow rate 12, 24 and 36 months after the end of radiotherapy under the circumstances that the protection of at least one single parotid is achieved either with 3D-CRT and IMRT. Depending on the radiation dose to the salivary glands the time of recovery of the parotid glands should be examined.

## Methods

### Patients selection

Between 06/2002 and 10/2008, 117 patients (90 male, 27 female, average age: 57 years) with squamous cell carcinoma of the head and neck were included in a prospective, non-randomised clinical study. These patients represent a cross-section of all patients receiving bilateral irradiation during tumor treatment of head and neck cancer at Martin-Luther-University Halle-Wittenberg (MLU-Halle), Germany. The tumors were classified in accordance with UICC TNM classification. All described schemes corresponded to the criteria of the official guideline. Patients' characteristics are described in table 1. The protocol was accepted by the ethics committee of the Martin-Luther-University Halle-Wittenberg. Study was supported by the German Cancer Aid e.V. The data in study Grant No. 106386 were prolonged in follow-up, and added to the data of the IMRT patients enrolled in study Grant No. 108429.

**Table 1:** Patient and tumor characteristics.

Study population	
Patient number	**117**

Male/female	**90/27**

Median age in years (range)	**57 (27 - 88)**

Unilaterale/bilaterale radiotherapy	**117**

3D-CRT/IMRT	**102/15**

**Tumour sites**	

Oral cavity/Oropharynx	**81**

Larynx/Hypopharynx	**29**

Unknown primary (CUP)	**2**

Other (Myeloma, Lymphoma, Nasal Cavity, Paranasal Sinus)	**5**

**Staging**	

UICC-I	**7**

UICC-II	**11**

UICC-III	**34**

UICC-IVA	**61**

UICC-IVB	**2**

Myeloma and Lymphoma IA/IIA	**2**

### Treatment planning, definition of target volumes and radiation dose

All patients received 3D-CRT or IMRT, the treatment of the bilateral neck was indicated, thus they were irradiated generally at primary tumor region and additionally regional lymph nodes.

Patients were immobilized with individual thermoplastic head-neck-shoulder masks. A computed tomography (CT) scan (General Electric Lightspeed, US) with slice thickness 5 mm of the head and neck region was performed for 3D-CRT or IMRT treatment planning.

**Figure 1:**
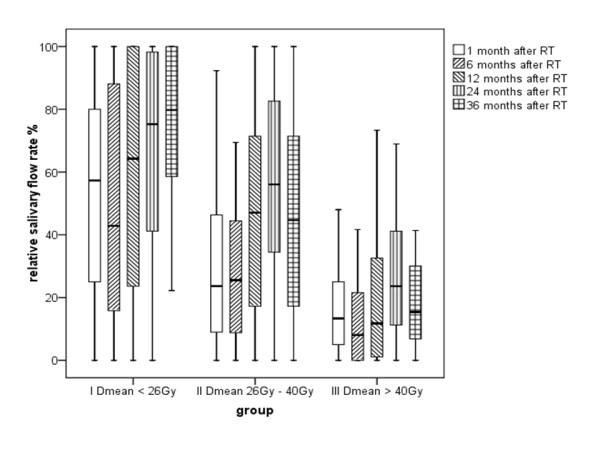
**Recovery potentials 1, 6, 12, 24, and 36 months after end of radiation therapy (RT)**. The initial flow-rate was 100%. Saliva measurement was normalized in relation to pre-treatment results in relative salivary flow rates (RSFR's in %).

The Helax TMS (Version 6.1) and Oncentra Masterplan (V1.5/3.0 Nucletron B.V., Veenendaal, NL) was used as 3D treatment planning system. The 3D-CRT was performed by standardized six to seven portals arrangements [10]. 6 - 10 MV photons of a linear accelerator were used (Primus or Oncor, Siemens Medical Solutions, Germany). IMRT was based on the step-and-shoot approach with seven or 9 equidistant 6 MV beams. The treatment technique was similar to the previously described one by Georg *et al*. [11]. The treatment planning system used was Oncentra Masterplan (V1.5/3.0, Nucletron B.V., Veenendaal, NL). The planning strategy was to cover 95% of the PTVs with 95% of the prescribed dose. The mean dose of at least one parotid gland was limited to 26 Gy without compromising the PTV, and the maximum dose to the spinal cord was 45 Gy, Figure 1.

Two different clinical target volumes (CTVs) were delineated: the CTV 1 harbouring the region of the primary tumor or postoperative tumor bed, including pathologically lymph nodes. The low dose volume was named CTV 2 and included the adjuvant treated regions of the neck without a histological or clinical proof of pathological changed lymph nodes. The primary planning target volume (PTV 1) was defined as CTV 1 with adequate safety margin of 5 mm. The secondary PTV (PTV 2) included PTV 1 and different lymph node chains of the neck (CTV 2) with a safety margin of 5 - 8 mm. The safety margin could be reduced close to the organs at risk. PTV 2 was irradiated five days a week, each fraction with a single dose of 2 Gy, until a cumulative dose of 50 Gy was reached. Afterwards PTV1 was continued to be irradiated in the same way until a total dose of 64 - 70 Gy. Dose specifications are related to a reference point in the target volume as described in ICRU reports 50, 62 and 83.

### Determination of the parotid gland doses

The planning target volumes and both parotid glands, the mandible, and the larynx were outlined on the transversal slices of the planning CT-scans. The planning goal was - while maintaining a homogeneous dose distribution in the target volumes - to minimize mean dose in the contra-lateral parotid gland. No effort was undertaken to spare the submandibular, the sublingual or minor salivary glands.

The mean dose and the partial volumes receiving specified doses were determined for each gland from dose-volume histogram (DVH). Based on an algorithm initially proposed by *Lyman *the DVHs, which represent non-uniform irradiation of the glands, were transformed to single step DVHs [12]. Afterwards, mean doses of the ipsilateral and contralateral parotid glands were calculated for every patient in Gy (D_mean_). The patients were grouped according to the D_mean _of the lowest irradiated parotid gland: Group I - D_mean _< 26 Gy (n = 36), group II - D_mean _26 - 40 Gy (n = 45), and group III - D_mean _> 40 Gy (n = 36).

### Determination of the saliva flow rate

All patients underwent saliva collection at different stages: within one week before radiation treatment, 1, 6, 12, 24, and at least 36 months after the end of RT. All salivary samples were collected at least one hour after a meal at a standardized time of the day (9:00 am to 11:00 pm). Patients were asked to rinse the mouth and swallow any residual saliva. Then, the patients were instructed to chew on a paraffin pellet (Ivoclar Vivadent^®^, Liechtenstein) for 5 min. After 5 min samples were collected with the patients expectorating all saliva into cups. Saliva was drawn up into one way syringes and salivary flow rates were expressed in millilitre (ml) per 5 min [13,14]. Saliva measurement was normalized in relation to pre-treatment results in relative salivary flow rates (RSFRs). In some cases patients produced a larger amount of saliva after radiotherapy than in the beginning. These measurements were regarded as free of complication and as 100 per cent of post therapeutic salivary flow rate.

### Statistics

The statistical analysis was performed using SPSS 17.0 for Windows. Direct comparisons (paired t tests) were used for the evaluation of differences in the lowest Dmean parotid dose and RSFRs. Comparison of salivary flow rates (RSFRs) and Dmean of the lowest parotid gland on months 12, 24 and 36 was accomplished by one-way ANOVA followed by post-hoc Bonferroni multiple-comparison test. Linear regressions were carried out on the results, assuming a normal distribution of the parameters D_mean _lowest parotid gland, tumor size, T and N stage and the correlation coefficients were determined. Level of significance was set to 5% (p < 0.05).

## Results

### Mean parotid gland dose of 3D-CRT and IMRT

15/117 (13%) patients received IMRT. In group I the number of patients with IMRT was 12/36 (33%), in group II 3/45 (13%) and in group III 0/36 (0%). By use of IMRT, the mean dose value of the spared parotid gland was significantly reduced compared to 3D-CRT (Table 2).

**Table 2:** Mean parotid gland doses with 3D-CRT and IMRT.

	IMRT	Patients n	Mean Dose (Gy)	Standard Deviation	p
**Spared (lowest) parotid** **gland**	No	102	34.4	13.6	0.001

**Spared (lowest) parotid** **gland**	Yes	15	21.7	6.2	

### Relative salivary flow rates

During the whole treatment course time the RSFRs decreased continuously and followed an exponential curve till 6 months after irradiation. The decline of RSFRs began directly after initiation of the radiation treatment. The reduction was already less pronounced in group I as compared to group II and particularly to group III (Table 3). Six months after radiotherapy the RFSR as compared to the initial flow rate was decreased to 50% in group I, 33% in group II, and 13% in group III. The comparison between group I and II did not demonstrate significant differences after 6, 12, 24 and 36 months. However, the comparison between group I and III did reveal significant differences at all re-examination time points (p < 0.05).

**Table 3:** Mean and standard deviation of relative salivary flow rate at 1, 6, 12, 24, and 36 months after radiotherapy.

Group		Patients (n)	mean RSFR (%)	SD (%)
I Dmean < 26 Gy	1 month after RT	34	55.6	32.71
	
	6 months after RT	35	50.2	36.44
	
	12 months after RT	27	59.7	36.46
	
	24 months after RT	19	65.8	34.10
	
	36 months after RT	14	74.3	27.85

II Dmean 26-40 Gy	1 month after RT	40	30.8	26.97
	
	6 months after RT	40	33.4	31.03
	
	12 months after RT	37	46.7	33.05
	
	24 months after RT	26	56.4	31.16
	
	36 months after RT	17	48.7	33.19

III Dmean > 40 Gy	1 month after RT	37	17.6	16.84
	
	6 months after RT	31	12.8	15.05
	
	12 months after RT	35	19.2	23.21
	
	24 months after RT	18	30.6	26.68
	
	36 months after RT	11	24.2	28.55

### Recovery of parotid glands

After 12, 24, and 36 months in group I and II a recovery effect could be measured. After 36 months, patients in group I had reached again about 74% of the initial value of saliva flow. The recovery during a follow up period of 24 months or 36 months was significant for group I and group II, whereas in group III no recovery potentials were measured neither at 12, 24, or 36 months (Figure 1).

### Impact of parotid dose, tumor site, tumor- and lymph node stage

Analysis of the RSFR as a function of the mean parotid dose between the different tumor sites (oral cavity, oropharynx, and larynx/hypopharynx), T stage and N stage was performed. A significantly greater parotid flow ratio after 36 months after RT in favour of the tumor sites larynx/hypopharynx (62%) and oropharynx (56%) as compared to oral cavity (31%) was shown (Table 4). Multiple linear regression analyses revealed that the parotid gland dose and the tumor site were the independent determinants 12 and 36 months after the end of RT (Table 5).

**Table 4:** Tumor site with mean and standard deviation of relative salivary flow rate at 1, 6, 12, 24, and 36 months after radiotherapy.

Tumor site		Patients (n)	mean RSFR (%)	SD (%)
Oral cavitiy	1 month after RT	29	34.3	32.42
	
	6 months after RT	29	32.7	35.63
	
	12 months after RT	28	35.9	37.54
	
	24 months after RT	14	43.4	37.84
	
	36 months after RT	13	31,0	28.20

Oropharynx	1 month after RT	51	30.2	27.67
	
	6 months after RT	47	27.4	29.87
	
	12 months after RT	41	34.1	33.56
	
	24 months after RT	30	51.3	34.75
	
	36 months after RT	16	55.9	39.70

Hypopharynx/Larynx	1 month after RT	28	42.7	32.32
	
	6 months after RT	27	42.6	34.50
	
	12 months after RT	29	53.1	31.90
	
	24 months after RT	17	59.6	28.94
	
	36 months after RT	11	61.6	29.99

**Table 5:** Multiple linear regression analyses for relative salivary flow rates (RSFRs) in the observation periods 12, 24 and 36 months.

Variables	RSFRs (%) of 12 months after RT (R^2 ^= 0.299)		RSFRs (%) of 24 months after RT (R^2 ^= 0.199)		RSFRs (%) of 36 months after RT (R^2 ^= 0.416)	
	**ß (SE)**	**p-value**	**ß (SE)**	**p-value**	**ß (SE)**	**p-value**

**D_mean _lowest parotid gland**	-1.187 (0.244)	**0.0001**	-0.736 (0.338)	**0.034**	-1.160 (0.395)	**0.006**

**Tumor site**	8.886 (2.815)	**0.002**	7.796 (4.178)	0.068	11.310 (4.640)	**0.021**

**T stage**	-2.429 (3.173)	0.446	-5.880 (4.671)	0.214	-10.047 (5.230)	0.064

**N stage**	-2.870 (2.143)	0.184	-2.499 (3.232)	0.443	0.866 (4.153)	0.836

## Discussion

Bilateral irradiation in patients with head and neck cancer leads to a dose-dependent change of salivary output and altered salivary composition [9,15,16]. Small salivary glands in oral cavity are a part of mucosal target volume. The submandibular glands just as sublingual glands reside in the midst of level I. Recently Wang *et al*. have shown that with modern IMRT a partial sparing of single submandibular gland is probably feasible [17]. By consequently performed radiation treatment of carcinomas of the oral cavity, the oropharynx, and the larynx/hypopharynx, the sparing of submandibular salivary glands can only be taken into consideration in special cases. Sparing of parotid glands as well as submandibular glands with dose reduction of mucous membranes seems to be the most effective way to prevent hyposalivation after treatment. 3D-CRT as well as IMRT do allow the generation of high dose gradients around target volumes, and thus to spare organs at risk inclusive mucous membranes. In previous investigations we have proven that sparing the parotid gland alone by using 3D-CRT produces less hyposalivation than a conventional radiation technique (2D-RT) [9].

Recent investigations have shown that more and more patients can take the advantage from more advanced RT techniques such as IMRT. In a multicenter randomized study (PARSPORT trial) the advantage of the parotid sparing by using IMRT technique as compared to conventional 3D-CRT in terms of clinical outcome was investigated [6]. The authors found that 12 months after treatment, 39% of IMRT patients suffered from dry mouth as compared to 74% of conventional RT. We also have found that by using IMRT, the mean dose value of the spared parotid gland was significantly reduced as compared to 3D-CRT. By using IMRT a mean parotid gland dose < 26 Gy was reached in 12/15 patients (80%), and a dose range > 26 to 40 Gy in 3/15 patients (20%). No patient with IMRT has had a mean dose of > 40 Gy within the spared parotid gland.

Currently, in the literature only limited data is available providing long-term salivary flow measurements over several years. Solely Braam *et al*. have demon-strated a recovery concerning a time period of 5 years [8]. Most of the other analysis did not cover more than 12 months [18,19]. In the present prospective analysis, we have shown results for a time period of 36 months recovery of the salivary glands. We assume that most of the recovery processes have been completed within this period. To demonstrate dose-related differences in the recovery potential of the parotid gland, we have divided our patients into 3 groups. Our separation with D_mean _< 26 Gy, 26 - 40 Gy and > 40 Gy was based on common reports from the literature and was done by reasons of comparison with previous investigations and particularly to complement our own objective measurements with the investigations of the quality of life after salivary gland protection [20,21].

As described in other studies hyposalivation can be prevented by restricting mean parotid gland doses to 26 - 30 Gy [15,22-24]. In our study, nearly one quarter of the patients did benefit from sparing the parotid gland by using 3D-CRT. With IMRT this was possible for 75% of the patients. Three years after irradiation 76% of the pre-treatment salivary flow can be preserved in this group. These results are excellent and highlight the significant advantage of IMRT as compared to the conventional 3D-CRT-technique [25,26]. With a mean parotid gland dose lower than 26 Gy, the recovery of salivary gland function reaches about 74% of the initial value at 3 years. Otherwise, patients with a mean parotid gland dose above 40 Gy did not show significant recovery values.

Our analysis of the flow ratio as a function of the mean parotid dose between the tumor sites oral cavity and larynx/hypopharynx did demonstrate a significantly higher parotid flow ratio in favour of the lower sites in the neck (larynx/hypopharynx) after radiotherapy. Significant differences over a time period of 12 and 36 months after end of RT were observed. The independent influence of T and N stage could not be demonstrated clearly also due to the limited number of patients 24 and 36 months after end of RT.

Taking into consideration the tumor localization, still one third of the patients received despite the use of 3D-CRT more than 40 Gy to the spared parotid gland. These patients suffered a total damage of salivary gland function after irradiation. In a further study, we have already shown that the remaining stimulated saliva in these patients is not able to maintain oral health due to its pH and its buffer capacity [20]. In fact it promotes dental caries [27]. Considering the low pH of 6.4, remineralisation is not possible any more, instead dentine and root areas are demineralised. Accordingly, dental prearrangements have to accommodate these circumstances.

We know of some weaknesses in our analysis. At 3 years after irradiation, of 117 initially included patients only a limited number of patients have been available for follow-up measurements, respective 14-17-11 patients in group I, II and III. This number of patients shows the reality concerning investigations of recovery effects of the salivary glands over a long time period. We also know that the method of the whole stimulated salivary flow rate measure resulting in a higher salivary flow rate compared to the more detailed examination techniques of parotid gland alone with Lashley cups. But the method is robust, easy to use and non-invasive, simulating a physiological situation and showed the smallest variability for measuring the salivary flow rate [13,22,28].

Also, we mention the expected anatomical changes of the parotid glands during the head and neck irradiation [29]. This is known from studies in centers with the use of helical tomotherapy. Due to weight loss and tumor shrinkage especially in head and neck patients the parotid gland is expected to get higher doses than predicted. Studies, whether these changes have a significant influence on the salivary flow rates are not available.

Hence, it has to be accepted that approximately three quarter of IMRT and only one third of 3D-CRT patient's benefit from salivary gland sparing by an increasing of the salivary flow rates do to 12 and 24 months after radiation. However, the aim of the radiation protocol used in this study was to preserve salivary flow rate as high as possible. Lack of saliva predisposes the development of atypical, unusual and rapidly progressive and aggressive dental decay [4,30,31].

## Conclusions

IMRT provides remarkable success rates as compared to conventional 3D-CRT in terms of parotid gland sparing. The IMRT technique should therefore represent the standard of care for the treatment of head and neck tumors. Parotid-gland-sparing up to mean doses of 26 Gy proved to be a reliable method to avoid distinct long lasting xerostomia.

## List of abbreviations

3D-CRT: Three-dimensional conformal radiotherapy; IMRT: intensity modulated radiotherapy; RT: Radiation therapy; RSFR: Relative Salivary Flow Rate; Dmean: Dose mean value; SD: Standard deviation; SE: Standard error; T: Tumor; N: Lymph node.

## Competing interests

The authors declare that they have no competing interests.

## Authors' contributions

JH gathered data and was the main author of the manuscript. JS performed statistical analysis. RG and MJ gathered treatment planning data. GH revised the manuscript and aided in the analysis. DV participated in the coordination. CG and TK conceived of the study, and participated in its design and coordination. All authors have approved the final manuscript.
